# Assessing National Antimicrobial Resistance Campaigns Using a Health Equity Assessment Tool (HEAT)

**DOI:** 10.3390/antibiotics8030121

**Published:** 2019-08-17

**Authors:** Graeme Hood, Lina Toleikyte, Diane Ashiru-Oredope

**Affiliations:** Public Health England, London SE1 8UG, UK

**Keywords:** antimicrobial stewardship, health inequalities, health equity assessment tool, public health

## Abstract

It has been widely recognised that a significant proportion of the world’s population suffer inequalities in accessing high quality healthcare and wider services. Within healthcare, antimicrobial resistance (AMR) is a global threat to public health affecting all healthcare systems and growing at an alarming pace. To ensure that national AMR campaigns developed by Public Health England are inclusive of all populations within the target audience a health equity assessment tool (HEAT) was used. The project leads for each campaign completed the HEAT independently with a follow up meeting with the study team to discuss and clarify the responses. A trend analysis was carried out with common themes being used to provide recommendations. The campaigns have demonstrated equality and diversity based on the requirements of the Equality Act 2010, particularly age, sex, and race protected characteristics. Some notable results include the translation of website materials in over 30 languages and reaching individuals in 122 countries. It was however noted that several of the protected characteristics were not applicable. The continuous development of resources with collaboration from a variety of diverse user groups would be advantageous towards aiding future campaign reach. The use of the HEAT has demonstrated the ease and cost-effective way to assess any health inequalities and would be a useful addition to antimicrobial stewardship and public health campaigns.

## 1. Introduction

Research has shown that there are inequalities in accessing high quality healthcare across the world, which affects a large proportion of people [1]. It has been estimated by the World Health Organisation (WHO) that 400 million people do not have access to one of seven essential health services, such as drugs and vaccines [2]. The variations in health inequalities have been reported both at a national and at an international level with the average life expectancy in the UK (81.2 years) lower than Japan (83.7 years) but higher than Sierra Leone (50.1 years) [1]. The health inequalities are classified as unjust differences in health and wellbeing between different groups of people which are systematic and avoidable and may be driven by (Supplementary Figure S1):

- Different experiences of the wider determinants of health or structural factors, for example the environments, income or housing.
- Differences in health behaviours or other risk factors between groups, for example smoking, diet, and physical activity levels have different social distributions. Health behaviours may be influenced by wider determinants of health, like income.
- Unequal access to or experience of health and other services between social groups.

The monitoring of health inequalities is important as it can identify progress linked to specific health policies or programmes and help to focus interventions at the most disadvantaged cohorts of the population. Through continual monitoring, the reduction or widening of health differences can be highlighted [3,4]. To aid the assessment of health interventions, policies, programmes, services and reduce health inequalities, specific assessment tools have been created such as the health equity assessment toolkit (HEAT) that was developed by the World Health Organisation (WHO) between 2014 and 2016 [5]. The WHO HEAT is a software, that contains the WHO’s health equity monitor database, supporting countries to assess inequalities within the country with over 30 indicators including reproductive, maternal, new-born and child health and five dimensions of inequality (economic status, education, place of residence, subnational region and child’s sex, where applicable) [5].

The reduction of health inequalities within England is a responsibility for Public Health England (PHE), an executive agency of the UK Government [6]. To help the organisation assess the impact of the public facing campaigns for health equity, PHE’s Health Inequalities team designed an internal HEAT. The assessment consists of a series of questions, which are designed to support systematic assessment of health inequalities related to work programmes, initiatives, policies and identify actions that need to be taken to reduce any potential identified inequalities.

The HEAT was built on international experience from the WHO and the Ministry of Health in New Zealand [5,7]. Unlike the WHO and New Zealand HEAT, the PHE tool considers the requirements of the Equality Act 2010 and therefore the set protected characteristics (Box 1) in addition to inequalities.

Box 1 **Protected characteristics (Equality Act 2010).**

- age
- sex
- race
- religion or belief
- disability
- sexual orientation
- gender reassignment
- pregnancy and maternity
- marriage and civil partnership

As well as considerations about socio-economic differences, area variations by the deprivation level (IMD), service provisions, urban/rural populations or in general and excluded and underserved groups for example homeless people, people in prison, or young people leaving care were considered.

It also recommends five stages for the assessment which include prepare, assess, refine, apply and review. Each assessment is likely to be iterative and can help areas continuously improve the contribution of work streams to reducing health inequalities. There have been notable local results within New Zealand from the application of this toolkit, including health promotion and health care clinics in low decile schools; dental care for those on low incomes; free interpreter services; and mobile Māori nursing services [8]. The WHO HEAT is pre-populated with the health equity monitor database, allowing users to explore the situation in one setting of interest (e.g., a country, province, or district) to determine the latest situation of inequality and changes in inequalities over time as well as to benchmark settings/countries. The PHE and New Zealand tools can be considered quality improvement tools for assessing and developing programmes, initiatives and policies.

It is important to use the HEAT to assess that the impact of public facing campaigns. The development of an internal HEAT has helped PHE to assess such interventions and provides a pragmatic and systematic set of steps for health policy makers to assess their initiative in relation to health equity. The application of the toolkit can occur at different stages of the program or policy development including the initial planning phase, the implementation stages or in the programme review phase [7]. Within the development of proposals or policies, there can be a tendency to leave the target groups as wide as possible so that more people are included. However, this approach can potentially result in the reduction of certain cohorts of the population that would benefit most from the programme or policy. This can result in unintended and unanticipated health impacts on certain population groups [9]. The use of HEAT can potentially help to reduce this unintended outcome for which PHE has started to incorporate this toolkit within the assessment process of national campaigns. The outcome of health equity assessments can inform decisions on how to build and strengthen policies, programmes and future services that organisations provide [8]. The antimicrobial resistance team has been one of the pioneers to adopt it within its campaigns.

Antimicrobial resistance (AMR) is a global threat to public health affecting all healthcare systems and growing at an alarming pace [10,11]. The implementation of antimicrobial stewardship programmes has been used within secondary care to help reduce this burden, with a focus on the UK Government ambition to reduce inappropriate antibiotic prescribing [12]. An important element of these programmes is the use of national campaigns to increase the overall reach. There is currently a lack of research assessing the potential health inequalities within public facing antimicrobial stewardship campaigns. The primary aim of this study was to assess whether the public facing AMR initiatives are reaching a diverse population within England. The secondary aim included providing individual campaign recommendations and general trend recommendations for the overall campaigns. Following a literature review, the authors believe there is currently limited research available that specifically assesses health inequalities related to AMR national campaigns.

### National Campaigns

The following four national AMR campaigns run by Public Health England were assessed:

Antibiotic Guardian

As part of the European Antibiotic Awareness Week (EAAD), this campaign was launched in September 2014. The aim was to increase commitment by healthcare professionals and members of the public to reduce AMR, change behaviour and increase knowledge. This is done through a pledge-based system where healthcare professionals and members choose a relevant pledge relating to their practice or personal situation [13].

Target audience: Broad audience

e-Bug

This campaign was developed as a European Union project and created a junior and senior school educational resource for teachers covering microbes, hygiene, antibiotics and the prevention of infection. There is both a paper based educational pack along with a web site that hosts games for young people and their families to play in the classroom or at home [14].

Target audience: School age children and teachers

TARGET (Treat Antibiotics Responsibly, Guidance, Education, Tools)

The TARGET antibiotics toolkit was developed by PHE in collaboration with the Royal College of General Practitioners (RCGP) and other professional societies. The main aim of this campaign is to improve antimicrobial prescribing in primary care through multiple channels such as guidance, interactive workshops, patient facing educational and audit materials [15]. 

Target audience: Prescribing clinicians

Keep Antibiotics Working

This is a consumer-led campaign to help raise awareness of antibiotic resistance to members of the public and highlight the dangers of not using antibiotics appropriately. 

There are three main aims within this campaign—alert the public to the issue of AMR, reduce public expectation for antibiotics and support change amongst healthcare professionals [16]. 

Target audience: Women aged 20–45 and Men and women aged 50+

## 2. Results

The overall results assessing whether each protected characteristic as defined in the Equality Act 2010 was addressed by the campaign are summarised in Table 1.

### 2.1. Antibiotic Guardian

The overall reach of this campaign was found to be extensive with 122 countries having at least one pledge [17]. This included the Antibiotic Guardian website currently being translated into 4 languages as well as English. When reviewing the population within England, it may be advantageous to offer languages in line with the latest Census (e.g., 2011 Census in England and Wales showed 7.7% of the population had another main language that was not English) [18]. The materials are open to everyone who has internet access so can be accessed by a variety of local populations and specific cultural groups. Further engagement and understanding of the culture of specific groups (e.g., Black, Asian and minority ethnic (BAME) and travelling community) or those in deprived communities would be beneficial to help promote the materials.

The Antibiotic Guardian campaign has now been embedded in the boy scouts to improve reach within younger children, an addition within the equivalent guides would help provide this key resource to more children.

The accessibility of information within the website is very good with an easy to use interface. Supportive resources, via leaflets and videos, provide a platform for those that do not have internet access. It could be beneficial to create and promote paper versions (PDF/word) of antibiotic guardian pledges that can be used by local campaign leads so that individuals without the internet can also pledge. The website supports a subtitle function on the home page video but a transcription of the video would improve accessibility. Future interface designs of the website could be done in conjunction with specific minority groups.

### 2.2. e-Bug

The e-Bug focuses on the education element of infections within children, young people and those hard to reach in the community. This interactive campaign has been translated into over 30 languages and has approved trainers throughout England, Wales and Northern Ireland which improves accessibility for these resources. The e-Bug trainer events should be targeted at areas with greater antibiotic use which have greater deprivation and ethnicity. It was found that all resources are freely available to download and print from the internet. The resources are developed for a range of ages and therefore abilities within the community. In the Beat the Bugs resources, each lesson plan has a range of activities to suit a range of abilities. The antibiotic Beat the Bug resources include a specific patient facing pictorial resource for the public with language or learning difficulties. There is an increased awareness within the project team of pictorial and foreign language resources, with a commitment to continue to reassess the language being used is inclusive of all, especially for disadvantaged and minority groups. There is also an increase focus in the implementation of resources in deprived areas using additional work force and targeted training to local clinical commissioning group staff.

An analytical review of the e-Bug website usage and views would help identify further development and improvement to reduce health inequalities and could be beneficial.

### 2.3. TARGET

The TARGET resources were found to be easily accessible to all healthcare professionals via an open access website. The distribution was extensive and resources can be accessed to permanent, training or temporary general practitioners (GPs). This was also the case for the out of hour GPs, though further publicity within this group would aid the reach. The use of community pharmacists in promoting the use of TARGET resources would help improve reach to patients. The TARGET website contains patient facing resources to share in consultations for respiratory tract infections (including a pictorial version) and urinary tract infections for patients under and over 65 years. These patient leaflets are available in the most common non-English languages spoken in the UK.

### 2.4. Keep Antibiotics Working

The quantitative research completed by the internal marketing team demonstrated a representative sample of the population within England, however due to the size of the sample, it was not possible or appropriate to break down the data by every protected characteristic. The choice of media via the TV ensured the campaigned reached a broad cross section of the population. During the campaign period, over 766,000 posters and leaflets were distributed to a range of partners including local authorities, health care centres and housing associations with 92% of GP practices in England engaged [19]. Prisons were also included within the distribution along with GPs who reach those from lower socioeconomic backgrounds. The advertising featured red and white pills which have no gender or racial bias. All campaign research, including campaign tracking, strategic and creative development research was carried out across all socioeconomic groups. The campaign materials were designed for all groups to understand and were C2DE—skilled working class, working class and non-working (the three lower social and economic groups in a society) inclusive. It would be recommended to conduct a review impact analysis of the campaign by age, sex and socioeconomic group, and where appropriate, change the campaign strategy based on this evidence. It was noted that more data is required to change the marketing or targeting specific populations but the use of advertising routes that are set up to engage with minority groups (e.g. radio channels focusing on certain groups, promote in religious areas, prisons) may be beneficial. It could be advantageous to translate resource leaflets into multiple languages in line with latest national Census and local population.

## 3. Discussion

The assessments highlight the diverse and inclusive nature of the four campaigns run by Public Health England. There have been notable successes in reaching a wider audience such as 92% of GP practices in England being engaged with the Keep Antibiotic Campaign. Interestingly, this wider reach may have contributed to a positive impact on the intended behaviour, with 78% of the public stating that they would be unlikely to ask their GP for antibiotics [19]. It is important to note that the reach of a campaign on the population can also have an influence on healthcare professionals with 93% of GPs saying they felt the materials supported them to say no to antibiotics when they were not needed [19]. As technology advances, it has been demonstrated that there is more of an emphasis being put on the use of technology within healthcare over the last few years. It is vital that campaigns use this to reach greater audiences but also remembering an ageing population who potentially do not have access to technology or would prefer not to be contacted through this means. The use of paper-based approaches are still of value and should be utilised to make sure it is inclusive of all.

The assessments were conducted by the project lead and team members which could cause bias when completing the assessment. However, through the use of an independent study team who are not actively involved within the projects, this was partially negated during the review meeting where the member of the study team provided a focus on the context of each question and did not aid or prompt the project lead around a particular response. It would be ideal to have an independent person completing the assessment without the involvement of the project lead but due to the complexities of the campaigns, specialist knowledge is required to provide meaningful results.

During the assessments, it became apparent that not all protected characteristics were appropriate for each campaign, for example, the e-Bug where the target audience is children, the marriage and civil partnership criteria for assessment was not applicable.

The way in which each campaign runs alongside each other is important with the integration and collaboration being key to improving the overall AMR message. The involvement of a senior sponsor of the proposed campaign during the review stage would aid this further integration and also make sure that organisational oversight is achieved.

The use of behavioural insight teams can also play a significant part in helping change an individual’s views when it comes to antibiotic use with a greater input in all campaigns would help achieve a long-term improvement when the public use antibiotics.

One of the limitations of this study is that the analysed/numeric data from the national campaigns are not presented. However, it is important to note that whilst these are not presented within the manuscript, the data and evidence are an essential part of section A—prepare the health equities assessment tool (HEAT) (S1). This study focuses its attention on the importance of national campaigns considering and monitoring health inequalities in their development and revisions. This will ensure that interventions do not widen health inequalities and also, they can be focused at the most disadvantaged cohorts of the population.

To improve the output of future campaigns, further research on health inequalities and AMR is needed. This would provide corresponding data to help tailor campaigns to specific individuals that may not be reached. Of the limited data available, there have been significant findings, including the increase of AMR among refugees and asylum seekers in high-migrant community settings [20]. This has highlighted where future policy could be implemented, but with this data being available it can also contribute to the effectiveness of using the HEAT in other health economies. Although this study only assessed each campaign once, the use of HEAT is an iterative process which can help to continuously improve the contribution of campaigns to reduce health inequalities. To consider the impact this assessment has in reducing health inequalities, a subsequent review is advised.

## 4. Materials and Methods

An internal HEAT (Supplementary Figure S1) developed by PHE was used. The project leads for each campaign in England, UK were asked to complete the HEAT independently, over an eight-week period from October to November 2018, with input from their team members as required. There was a 100% (4/4) completion rate to the HEAT during the allocated period. An initial meeting was conducted with the project lead and one member of the study team to discuss the rationale of the assessment tool and explain how the tool should be used. The HEAT was sent to each project lead via email with responses also returned in the same way. A follow up meeting between the same individuals was arranged to discuss and clarify queries and to standardise the approach for each separate assessment. This was done to be able to provide trend analysis over all the assessed campaigns. All the responses were collated by one member of the study team with a summary of the responses being discussed with the remaining members of the study team. The main focus of the overall analysis was assessing whether each protected characteristic as defined in the Equality Act 2010 and is the focus of the PHE HEAT was included within the individual campaigns. A trend analysis was carried out on all four campaign assessments with common themes of the responses being used to provide recommendations for future work. A common theme was confirmed if two or more of the campaign responses identified that theme. A qualitative analysis was used to summarise each campaign against the protected characteristics. An action plan for each project was developed inclusive of common themes but also of any specific recommendations relevant to that campaign.

## 5. Conclusions

The use of the HEAT comes at a significant time when the threat of AMR is well publicised. Making sure any stewardship campaign is as diverse and inclusive as possible is essential to improve the reach of the target audience. The greater the reach, the more impact national campaigns have with greater flexibility to make them accessible for a diverse population. To help tailor campaigns to hard to reach groups, further research into health inequalities relating to accessing antimicrobials would be beneficial. The assessment has shown to be an easy and cost-effective way to assess any health inequalities within a campaign and could be a useful addition to local level stewardship programmes that are trying to reduce the amount of inappropriate antimicrobial use. This systematic approach helps facilitate opportunities to address any health inequalities that have been identified and can also be applied to a range of services and programmes. The communication of information within diverse populations is vital in reducing the burden of AMR and addressing health inequalities. To improve the reach of this information, the use of HEAT is also vital.

## Figures and Tables

**Table 1:** Campaign assessment of the protected characteristics.

Protected Characteristic	Antibiotic Guardian	e-Bug	Target	Keep Antibiotics Working
Age	All ages	Ages 9–24	All ages	Ages 20–45 and 50+
Sex	All	All	All	All
Race	Website available to all	Translated into over 30 languages	Translated into approximately 20 languages	Resources distributed throughout the UK
Religion or Belief	No specific information	No specific information	No specific information	N/A
Disability	No specific information	No specific information	Material for learning disability users	Material designed for all groups to understand (C2DE inclusive)
Sexual Orientation	N/A	N/A	N/A	N/A
Gender reassignment	N/A	N/A	N/A	N/A
Pregnancy and maternity	No specific information	N/A	Information on pregnancy within leaflets	Aimed at females responsible for family health
Marriage and civil partnership	N/A	N/A	N/A	N/A

N/A—no data available.

## Supplementary Materials

The following are available online at https://www.mdpi.com/2079-6382/8/3/121/s1, Figure S1: The PHE Health Equity Assessment Tool. * This assessment tool is currently a working draft that the PHE’s Health inequalities team is planning to revise in line with stakeholder feedback.

## References

[1] Global Health Inequalities. https://researchbriefings.parliament.uk/ResearchBriefing/Summary/POST-PN-0553.

[2] Tracking Universal Health Coverage: First Global Monitoring Report. https://www.who.int/healthinfo/universal_health_coverage/report/2015/en/.

[3] WHO Handbook on Health Inequality Monitoring with a Special Focus on Low and Middle Income Countries. http://www.who.int/gho/health_equity/handbook/en/.

[4] State of Inequality: Reproductive, Maternal, Newborn and Child Health. http://www.who.int/gho/health_equity/report_2015/en/.

[5] Hosseinpoor A.R., Nambiar D., Schlotheuber A., Reidpath D., Ross Z. (2016). Health Equity Assessment Toolkit (HEAT): Software for exploring and comparing health inequalities in countries. BMC Med. Res. Methodol..

[6] Public Health England About Us. https://www.gov.uk/government/organisations/public-health-england/about.

[7] The Health Equity Assessment Tool: A User’s Guide. https://www.health.govt.nz/publication/health-equity-assessment-tool-users-guide.

[8] Sheridan N.F., Kenealy T.W., Connolly M.J., Mahony F., Barber P.A., Bpyd M.A., Carswell P., Clinton J., Delvin G., Doughty R. (2011). Health equity in the New Zealand health care system: A national survey. Int. J. Equity Health.

[9] Equity Focussed Health Impact Assessment Framework. http://hiaconnect.edu.au/old/files/efhia_framework.pdf.

[10] WHO Antimicrobial Resistance. http://www.who.int/mediacentre/factsheets/fs194/en/.

[11] Review on Antimicrobial Resistance. https://amr-review.org/background.html.

[12] Research Reveals Levels of Inappropriate Prescriptions in England. https://www.gov.uk/government/news/research-reveals-levels-of-inappropriate-prescriptions-in-england.

[13] Keston J.M., Bhattacharya A., Ashiru-Oredope D., Gobin M., Audrey S. (2017). The Antibiotic Guardian campaign: A qualitative evaluation of an online pledge-based system focused on making better use of antibiotics. BMC Public Health.

[14] McNulty C.A., Lecky D.M., Farrell D., Kostkova P., Adriaenssens N., Koprivova Herotova T., Holt J., Touboul P., Merakou K., Koncan R. (2011). e-Bug Working Group. Overview of e-Bug: An antibiotic and hygiene educational resource for schools. J. Antimicrob. Chemother..

[15] Jones L.F., Hawking M.K.D., Owens R., Lecky D., Francis N.A., Butler C., Gal M., McNulty C.A.M. (2017). An evaluation of the TARGET (Treat Antibiotics Responsibly; Guidance, Education, Tools) Antibiotics Toolkit to improve antimicrobial stewardship in primary care—Is it fit for purpose?. Fam. Pract..

[16] Keep Antibiotics Working. https://campaignresources.phe.gov.uk/resources/campaigns/58-keep-antibiotics-working/Overview.

[17] Newitt S., Oloyede O., Puleston R., Hopkins S., Ashiru-Oredope D. (2019). Demographic, Knowledge and Impact Analysis of 57,627 Antibiotic Guardians Who Have Pledged to Contribute to Tackling Antimicrobial Resistance. Antibiotics.

[18] Language in England and Wales: 2011. https://www.ons.gov.uk/peoplepopulationandcommunity/culturalidentity/language/articles/languageinenglandandwales/2013-03-04.

[19] English Surveillance Programme for Antimicrobial Utilisation and Resistance Report. https://assets.publishing.service.gov.uk/government/uploads/system/uploads/attachment_data/file/759975/ESPAUR_2018_report.pdf.

[20] Nellums L.B., Thompson H., Holmes A., Castro-Sanchez E., Otter J.A., Norredam M., Friedland J.S., Hargreaves S. (2018). Antimicrobial resistance among migrants in Europe: A systematic review and meta-analysis. Lancet Infect. Dis..

