# Supplementary material for: Assessing National Antimicrobial Resistance Campaigns Using a Health Equity Assessment Tool (HEAT)
Source: Antibiotics (Basel). 2019 Aug 17;8(3):121. doi: 10.3390/antibiotics8030121 (PMC6784169; doi:10.3390/antibiotics8030121)
Supplement: Supplementary file 1 [file antibiotics-08-00121-s001.pdf]

Supplementary Figure S1: The PHE Health Equity Assessment Tool

*This assessment tool is currently a working draft that the PHE's Health inequalities team is planning to revise in line with stakeholder feedback.

This tool consists of a series of questions, which are designed to help you systematically assess health inequalities related to your work programme and identify action that you can take to help reduce inequalities. It will also help you to consider the requirements of the Equality Act 2010.

The tool is designed to be used at the start of a work plan to help you consider its potential effects, but it can be used retrospectively. There are five stages – **Prepare, Assess, Refine, Apply, and Review** – but you can use the tool flexibly. In practice, your assessment is likely to be iterative and will help you continuously improve the contribution of your work to reducing health inequalities. It is important that a senior leader sponsors the assessment and is involved at the review stage.

Assessing health inequalities

Health inequalities are unjust differences in health and wellbeing between different groups of people which are systematic and avoidable. Health inequalities in England exist across a range of dimensions or characteristics, including some of the nine protected characteristics of the Equality Act 2010, socioeconomic position and geography (Box).

Health inequalities may be driven by:

1. Different experiences of the wider determinants of health or structural factors, for example., the environments, income or housing

Box: Examples of dimensions or characteristics to consider

Protected characteristics (Equality Act 2010):

- age
- sex
- race
- religion or belief
- disability[1]
- sexual orientation
- gender reassignment
- pregnancy and maternity
- marriage and civil partnership

Socio-economic differences by individual socio-economic position e.g. NS-SEC, employment status, income, area deprivation

Area variations by deprivation level (IMD), service provision, urban/rural or in general

Excluded and underserved groups for example homeless people, people in prison, or young people leaving care

2. Differences in health behaviours or other risk factors between groups, for example smoking, diet, and physical activity levels have different social distributions. Health behaviours may be influenced by wider determinants of health, like income.
3. Unequal access to or experience of health and other services between social groups.

People who share protected characteristics, defined in the Equality Act 2010, may experience poor health outcomes as a direct result of discrimination or due to different experiences of the factors described above.

We are interested in all of these drivers of inequality, as they apply to particular programmes or work strands but we recognise that there may be less scope to comment on some of them when considering specific work plans.

Health Equity Assessment

**Programme or project being assessed:**	
**Date completed:**	
**Contact person (Name, Directorate, email, phone)**	

Steps to take	Answers that relate to the work you are assessing. Remember to consider multiple dimensions of inequalities shown on page 1, including the protected characteristics, and socio-economic position.
**Steps to take**	**Answers that relate to the work you are assessing.** Remember to consider multiple dimensions of inequalities shown on page 1, including the protected characteristics, and socio-economic position.
**A. Prepare – agree the scope of work and assemble the information you need**	
**1. Aims**<br>- What are the main aims of your work?	➢ *Describe your initial work plans or intervention plans detailing the key outcomes to be achieved*<br><br>Your answer:

Steps to take	**Answers that relate to the work you are assessing.** Remember to consider multiple dimensions of inequalities shown on page 1, including the protected characteristics, and socio-economic position.
**2. Focus on health inequalities**<br>- How do you expect your work to contribute to reducing health inequalities	➢ *Consider whether the work is general or targeted/tailored to specific groups. How much does is explicitly focus on how to help improve outcomes or experiences for more disadvantaged groups? This will provide a reference point for understanding how to improve your focus on reducing health inequalities.*<br><br>Your answer:
**3. Data and evidence**<br>- What are the key sources of data and evidence that you need to identify health inequalities in your work?	➢ *To complete the remainder of this assessment, you will need to assemble background information on distributions of the issue or outcomes you are examining. Collate all information in one place so colleagues working in the same field can access it.*<br>➢ *You may need to commission a specific literature search to identify relevant evidence.*<br>➢ *You may find it helpful to include references or links to key sources here.*<br><br>Your answer:

Steps to take	**Answers that relate to the work you are assessing.** Remember to consider multiple dimensions of inequalities shown on page 1, including the protected characteristics, and socio-economic position.
**B. Assess – examine the evidence and intelligence**	
**4. Distribution of health**<br>- What health inequalities exist in relation to your work?<br><br>o Who is most advantaged and how?<br><br>o Who is most disadvantaged? Which groups have the poorest health outcomes or face discrimination in relation to your work or intervention?	➢ *Consider the different dimensions of health inequalities that exist in relation to your work –* e.g., *socioeconomic or geographic, or specific socially excluded groups*<br>➢ *Consider poor outcomes and experience related to protected characteristics*<br><br>Your answer:
**5. Causes of inequalities**<br>- What key factors drive differences in outcomes between groups? To what degree can you influence them?<br><br>o Which wider determinants are influential?<br>o What health behaviours play a role?<br>o To what degree do service or intervention experiences (e.g., issues of access or take	*As appropriate to your, try to consider*<br>➢ *the wider determinants of health or– e.g.,: education, employment and housing. You might be able to work through specific settings or inform relevant policy measures*<br>➢ *Inequalities in some conditions or diseases are linked to the social distribution of unhealthy behaviours or other risk factors so you might find it helpful to work with other teams.*<br>➢ *Service quality, take up and engagement – we know that meaningful community engagement is fundamental for providing high quality services that reach everyone.*<br><br>Your answer:

**Steps to take**	**Answers that relate to the work you are assessing.** Remember to consider multiple dimensions of inequalities shown on page 1, including the protected characteristics, and socio-economic position.
up) have implications for health inequalities in your work area or intervention?	
**C. Refine – focus on the actions that have the greatest impact**	
**6. Potential effects**<br>In light of the consideration you have now given to your programme or project assess the potential impacts.	*Consider the distribution of health outcomes and the cause of inequalities that you identified in previous questions.* ***Are your plans relevant to this evidence?***<br>➢ *Will/do your key outcomes vary across groups?*<br>➢ *Who benefits most? Who benefits least?*<br>➢ *What might the unintended consequences be?*<br>➢ *To what degree will your work reach people with greater need?*<br>.
a) How is the work likely to affect health inequalities? (positively or negatively)<br><br>b) How might your work address the needs of different groups that share protected characteristics?	Your answer:
**7. Adapt work plans or programme design**	➢ *Bear in mind any additional or complementary activities across your work programme that might fill any gaps that you find in your particular work stream.*<br>➢ *Think about all potential options – you will narrow down on a particular course of action later.*

Steps to take	**Answers that relate to the work you are assessing.** Remember to consider multiple dimensions of inequalities shown on page 1, including the protected characteristics, and socio-economic position.
a) What changes to your plans would increase the focus on health inequalities or achieve larger reductions in inequalities?<br>b) What changes to your plans would increase the focus on addressing the needs of groups that protected characteristics and advancing diversity and inclusion?	Your answer:

D. Apply – make tangible changes to work plans, informed by evidence

**8. Action plan**<br>In light of your assessment, what specific actions will you now undertake to strengthen your contribution to<br>a) reducing health inequalities?<br>b) promoting equality and diversity across groups that share protected characteristics?	➢ *Work to create or revise existing resources* e.g., *evidence/data resources, implementation tools or guidance*<br>➢ *New partnerships to influence policy or practice* e.g., *working with specific VCS bodies*<br>➢ *Specific evaluation or monitoring work*<br>➢ *Specific service or programme objectives* e.g., *KPIs*
**9. Evaluation and monitoring**	➢ *How far are you able to consider the effects on different groups? Are you able to evaluate whether differences increased or reduced over time during the course of your work stream or intervention?*<br>➢ *Consider whether there a need for a particular focus on any protected characteristics.*

Steps to take	**Answers that relate to the work you are assessing.** Remember to consider multiple dimensions of inequalities shown on page 1, including the protected characteristics, and socio-economic position.
How will you monitor and evaluate the effect of your work on:<br>a) Health inequalities<br>b) Promoting equality, diversity and inclusion	

**Now set a health equity assessment review date, recommended for between 6 and 12 months from initial completion.**<br>**Review date:**

**E. Review – identify learn lessons and drive continuous improvement**	
**Date completed:**	
**Contact person (Name, Directorate, email, phone)**	

Steps to take	**Answers that relate to the work you are assessing.** Remember to consider multiple dimensions of inequalities shown on page 1, including the protected characteristics, and socio-economic position.
**10. Lessons learned**<br>Have you achieved the actions you set?	

**Steps to take**	**Answers that relate to the work you are assessing.** Remember to consider multiple dimensions of inequalities shown on page 1, including the protected characteristics, and socio-economic position.
To what degree and in what ways have your work plans<br>a) supported reductions in health inequalities?<br><br>b) promoted equality, diversity and inclusion across groups that share protected characteristics.	
**Now, review other parts of the assessment and update them if necessary.**	
